# CT Findings and Clinical Features of Pancreatic Hemolymphangioma: A Case Report and Review of the Literature

**DOI:** 10.1097/MD.0000000000000437

**Published:** 2015-01-26

**Authors:** Liang Pan, Gao Jian-bo, Pullas Tapia Gonzalo Javier

**Affiliations:** From the Department of Radiology (LP, GJ-b), The First Affiliated Hospital, Zhengzhou University, Zhengzhou, China; and Department of Vascular and Endovascular Surgery (PTGJ), Military Hospital, Quito, Ecuador, South America.

## Abstract

Pancreatic hemolymphangioma is a very rare benign tumor. There were only 10 reports of this disease until June 2014.

The aim of the present study was to describe a hemolymphangioma in the neck and body of the pancreas in a 57-year-old woman.

The method used in the present study consists of description of the clinical history, image lab features, and pathological result.

The patient complained of a 10-day history of epigastric discomfort. Abdominal computed tomography (CT) showed a cystic–solid tumor with an irregular shape, in the neck and body of the pancreas. The tumoral cystic wall and its internal division could be seen intensified on contrast-enhanced CT images compared with those on precontrast images. The pathological examination confirmed the diagnosis.

The clinical feature of pancreatic hemolymphangioma includes a lack of specificity. The CT appearance combined with age and sex may be useful in making an early diagnosis.

## INTRODUCTION

Hemolymphangioma is a congenital malformation of the vascular system that is composed of cystically dilated lymphatics.^1,2^ It is preferentially located in the head and neck,^2^ seldomly in the spleen, small intestine, and vermiform appendix. The incidence of pancreatic hemolymphangioma is extremely rare. To the best of our knowledge, the total number of patients of pancreatic hemolymphangioma is only 10 in the revised literature.^1,3,4^ Almost all tumors were exclusively located in the head of pancreas, and there has been no report of any tumor in the neck and body of the pancreas. The diagnosis of hemolymphangioma remains controversial, as it may be confused radiologically with other cystic–solid or cystic lesions of the pancreas. In the present study, we analyzed the computed tomography (CT) characteristics and the clinical features of a rare case of pancreatic hemolymphangioma with a review of the literature.

## CASE REPORT

### Patient Information

A 57-year-old woman presented with a 10-day history of epigastric discomfort. She did not complain of nausea, vomiting, fever, weight loss, or night sweating. She had a history of hepatitis, sulfonamide allergy, and dexamethasone allergy.

### Diagnostic Focus and Assessment

**FIGURE 1:**
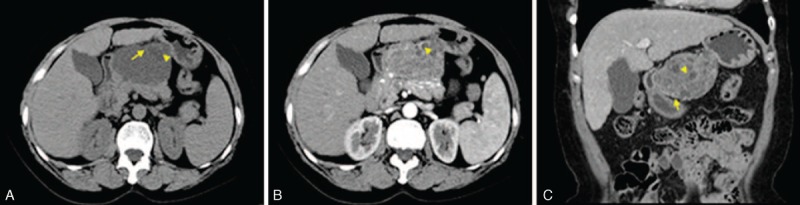
Abdominal CT demonstrating a heterogenous mass (arrow) at the neck and body of the pancreas, adjacent to the descending portion of duodenum and the lesser curvature of the gastric body. The cystic part has internal division change (arrowhead) (A). The mass, cystic wall (arrowhead), and internal division (arrow) could be seen intensified on contrast-enhanced CT images compared with those on precontrast images (B and C). CT = computed tomography.

A physical examination showed mild pain in the left hypochondrium without rebound tenderness. Laboratory blood tests were as follows: red cell count 3,560,000/L, neutrophil count 140,000/L, and platelet count 7500,000/L; neutrophils accounted for 37.5%, and lymphocytes accounted for 54.5%. The result of the hepatitis C virus antibody test was positive. No abnormalities were revealed except for carcinoembryonic antigen (1.03 ng/mL) in the elevated tumor markers. The levels of electrolytes and function tests of renal and liver were within normal limits. Abdominal CT showed a cystic–solid tumor (77.7 × 60.3 mm) with an irregular shape in the neck and body of the pancreas, adjacent to the descending portion of duodenum and the lesser curvature of the gastric body (Figure 1A). The tumoral cystic wall and its internal division could be seen intensified on contrast-enhanced CT images compared with those on precontrast images (Figure 1B and C).

### Therapeutic Intervention and Follow-Up

The patient was then transferred to our Gastrointestinal Surgery Unit in September 2013. The patient underwent a wide local resection of the tumor. After complete incision of the gastrocolic ligament, the mass was identified as a large dark red mass located at the neck and body of the pancreas, adjacent to the descending portion of duodenum and the lesser curvature of the gastric body, with bloody fluid inside.

**FIGURE 2:**
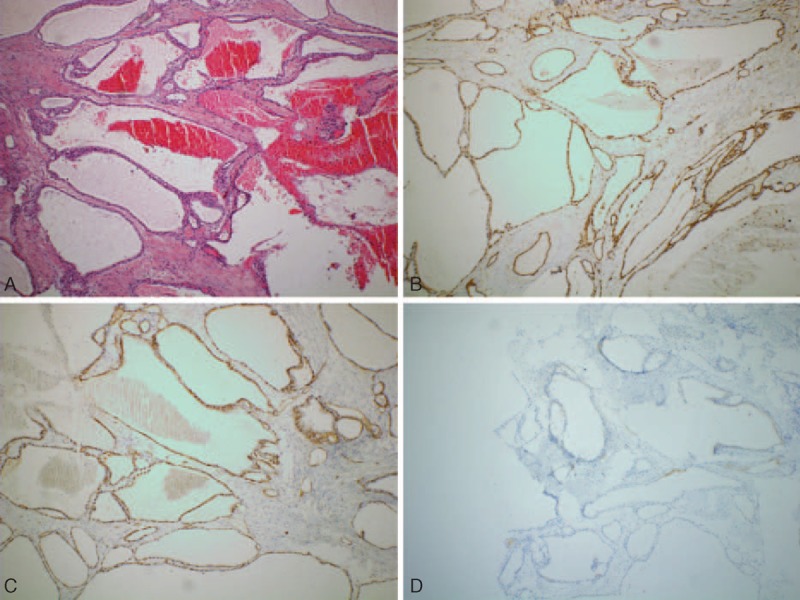
Histological analysis revealed a soft tissue mass, consisting of lymphatic and blood vessels (hematoxylin and eosin, ×100) (A). Positive immunostaining for CD31 (B) and CD34 (C) with positive staining for D2-40 (D) supported the diagnosis of pancreatic hemolymphangioma.

Macroscopically, the lesion was about 80 × 60 × 45 mm in diameter, and was a soft, polypoid mass with irregular lobulated margin, with lymphatic and blood vessels inside (Figure 2A). CD31 (Figure 2B) and CD34 (Figure 2C) were relatively positive in the cytoplasm of vascular endothelial cells. D2-40 revealed a relatively positive cytoplasm of lymphocyte cells (Figure 2D). Final pathological diagnosis of this tumor was a pancreatic hemolymphangioma.

After 2 months of follow-up by CT and ultrasonography, no complication or recurrence was observed.

### Literature Review

We searched PubMed, Medline, Google Scholar, Chinese Biomedicine Database, and the China Journal Full Text Database without language restriction. The search terms included (Pancreatic [MeSH]) AND Hemolymphangioma [MeSH]). We initially identified 10 relevant items in PubMed, Medline, Google Scholar, Chinese Biomedicine Database, and the China Academic Journals Full-Text Database. Publication dates ranged from 1997 to June 2014. After reviewing each publication, we selected 10 original studies. The characteristics of 9 patients with hemolymphangioma of the pancreas are shown in Table 1.

**TABLE 1:**
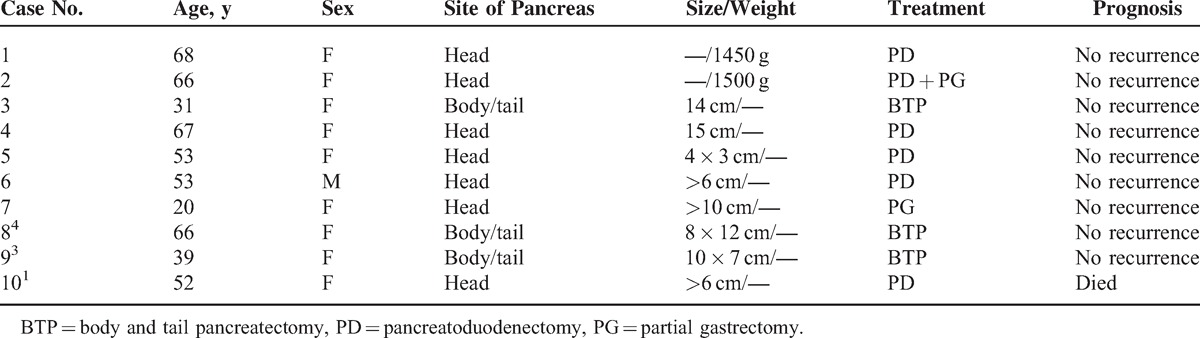
Characteristics of 10 Patients With Hemolymphangioma of the Pancreas^1,3–5^

## DISCUSSION

Hemolymphangioma, also known as the vascular hamartoma, is an extremely rare congenital malformation of the vascular system that is composed of cystic dilated lymphatics.^1,2^ It is preferentially situated in the head and neck,^2^ seldomly in the spleen, small intestine, and vermiform appendix. The formation of this tumor may be explicated by obstruction of the venous–lymphatic communication among dysembryoplastic vascular tissues and the systemic circulation.^1,3,5–9^ Pancreatic hemolymphangioma was originally described by Couinaud et al in 1966, and several reports have since been published in the literature. Nevertheless, it remains a rare cystic benign tumor of the vascular tumors of the pancreas, representing 0.1% of primary pancreas tumors.^5,6^ In a review of published work until June 2014 (PubMed, Medline, Google Scholar, Chinese Biomedicine Database, and the China Journal Full Text Database), we found only 10 case reports.^1,3–5^

Pancreatic hemolymphangioma typically arises in adult patients ranging in age from 20 to 68 years, with an average age of 51.5 years. Almost 70% of described cases occurred in patients over the age of 51.5. Pancreatic hemolymphangioma is most common in female patients, with the sex distribution of male to female 1:9.^1,3,4^ The clinical manifestation is not typical, whereby it can be hidden for a long time. Abdominal pain and epigastric discomfort are the most common symptoms,^1,3,5^ and some other nonspecific symptoms such as vomiting and nausea are caused by external gastric compression.^5^ The symptom of severe anemia, caused by duodenal bleeding due to the pancreatic hemolymphangioma invaded to the duodenum, is extremely rare.^6^ Laboratory tests of pancreatic hemolymphangioma are frequently normal, although the case reported by Banchini et al had a slight increase in alkaline phosphatase and γ-glutamyl transferase.^8^

Pancreatic hemolymphangiomas are usually large lesions with a diameter of larger than 10 cm, and almost all tumors were exclusively located in the head of pancreas.^1,5^ A survey of the literature available showed that only 3 cases were found in the body and tail of the pancreas.^9^ To the authors’ knowledge, there has been no report of pancreatic hemolymphangioma located in the neck and body of the pancreas. Imaging has been frequently used to make the diagnosis of cystic tumors of the pancreas and follow-up of the patients with pancreatic hemolymphangioma. Currently, endoscopic ultrasonography is the most commonly used, economical, and simple means to clinically diagnose pancreatic diseases. Typical endoscopic ultrasonography findings of pancreatic hemolymphangioma are anechoic mass with clear edge.^1^ However, in the present study, this diagnostic option was not available. CT and magnetic resonance imaging (MRI) are other usual methods for the diagnosis of pancreatic hemolymphangioma. They are usually performed to investigate a possible communication of the mass with the pancreas and to further characterize the mass. On MRI, the findings of pancreatic hemolymphangioma such as lymphangiomas and most vascular malformations typically show a cystic–solid mass with hyperintense signal intensity on T2-weighted and turbo short T1 inversion recovery images.^1^ CT findings of the tumor usually presented as cystic–solid masses that have a single large cyst and a small solid part. The cystic part might be caused by the rupture and fusion of the vascular cavity and the solid part represented the residual and compressed vascular tissue.^3^ In the present case, the tumor appeared as a multiloculated cystic mass with slight enhancement in cystic wall and internal division. The histopathological examination suggests that the tumor consists of abnormal lymphatic and blood vessels with polycystic spaces, and the thin-walled cystic lesion has connective septa covered by endothelium. Relatively few studies have been done to investigate the CT contrast enhancement features of pancreatic hemolymphangioma, and, again, the opinions were controversial in most studies. Sun et al^5^ reported a 20-year-old girl with diagnosis of pancreatic hemolymphangioma with partial blood flow in abdominal cavity. This tumor manifested distinct boundary, which was intensified obviously in portal venous phase, whereby it is suggested that CT contrast enhancement scan may improve the detection rate. However, it is worth mentioning that a previous study has reported a pancreatic hemolymphangioma presented as a cystic–solid tumor with no enhancement in the 3-phase contrast scan, which may be related to the relatively small number of blood vessels in the tumor and slow-moving blood flow in the dysplastic blood vessels.^3^

The tomographic diagnostic of pancreatic hemolymphangioma is not often, due to the difficulty to reach a correct differential diagnosis of other cystic–solid or cystic lesions of the pancreas before surgical resection. Mucinous cystadenoma is predominantly detected in middle-aged females, and almost all tumors are exclusively located in the body and tail of pancreas. It is usually a single large cyst with a diameter of more than 2 cm, smooth border, fine septa, even wall, and potentially, small nodules. The appearance of the tumor diameter more than 8 cm, solid part increased in cyst, or thin or thick cystic wall and septum, and large nodules, or calcification are highly suggestive of cystadenocarcinoma. In general, differential diagnosis through CT image of mucinous cystadenoma with cystadenocarcinoma is difficult because both of them can present septa, walls, and nodule enhancement. Pancreatic pseudopapillar solid tumor is typically found in young women, which is a borderline malignant potential tumor. Almost all tumors present a cystic–solid mass with a clear border, with or without calcification, seldom seen dilations of biliary and pancreatic duct, with enhancement of the solid part. Pancreatic pseudocyst is typically a unilocular lesion and cyst wall thickening with or without enhancement, and other features including peripancreatic fat fuzzy interval, prerenal fascial thickening, etc, with a history of pancreatitis. However, the definitive diagnosis of pancreatic hemolymphangioma is based on a combination of clinical, radiological, and histopathological evidences.^3,7^

Despite hemolymphangioma being commonly a benign disease, there is a possibility of recurrence and invasion of surrounding organs.^3,6^ The surgical resection appears to be the most effective treatment, especially when the tumor increases in size and applies pressure on the surrounding tissues. The range of surgery usually includes complete removal of the tumor with the surrounding organs that may be potentially invaded. Toyoki et al^6^ reported a 53-year-old man with severe anemia, caused by duodenal bleeding due to tumoral invasion. Dong et al^3^ reported a 20-year-old girl with pancreatic hemolymphangioma, who complained of a giant mass in abdominal cavity and epigastric discomfort. The tumor infiltrated the transverse mesocolon and greater omentum, and was tightly adhered to the duodenum and superior mesenteric artery. The recurrence rate varies depending on the complexity, the anatomical location, and the adequacy of the excision.^2^ It has been established in the literature that lesions that have been completely excised show 10% to 27% recurrence, while 50% to 100% of partly resected tumors may recur.^2^ However, no recurrence and invasion of pancreatic hemolymphangioma had been reported previously. Tumor removal may also be associated with complications such as infection, fistula, and hemorrhage.^2,5^

The clinical feature of pancreatic hemolymphangioma includes a lack of specificity. The CT appearance combined with age and sex may be useful in making an early diagnosis. Despite it being commonly a benign disease, there is a possibility of recurrence and invasion of surrounding organs. Surgical resection is required to treat this tumor; careful follow-up with CT or ultrasound is necessary.
